# Self-mutilation of Fingers Following Median Nerve Injury: Case Reports and Literature Review

**DOI:** 10.7759/cureus.7872

**Published:** 2020-04-28

**Authors:** Wafa Binfadil, Rahul P Sinha, Hayder Saleh, Farhan Ali, Sattar Alshryda

**Affiliations:** 1 Trauma and Orthopaedic, Rashid Hospital, Dubai, ARE; 2 Neurology, Al Jalila Children's Speciality Hospital, Dubai, ARE; 3 Orthopaedic, Rashid Hospital, Dubai, ARE; 4 Orthopaedic, Royal Manchester Children Hospital, Manchester, GBR; 5 Pediatric Orthopaedics and Trauma, Al Jalila Children's Speciality Hospital, Dubai, ARE

**Keywords:** supracondylar humeral fracture, median nerve injury, self-mutilation, self-biting, children, nerve injury

## Abstract

Self-mutilation of fingers following nerve injuries is extremely rare, but it can lead to serious complications, including amputation if not treated timely. We report here what we believe to be the first English language reports of self-mutilation of fingers following median nerve injury caused by a supracondylar humeral fracture.

## Introduction

Elbow fractures are common in children representing 5-10% of all fractures with supracondylar fractures of the humerus (SCH) being the most common type [1, 2]. Based on the direction of fracture angulation, SCH fractures are classified into extension type (the commonest) and flexion type. The distal fragment angulates posteriorly in the former and anteriorly in the latter. The commonest mechanism of injury is falling on an outstretched hand, where the elbow is hyperextended, the tip of the olecranon process is wedged through the olecranon fossa, and the anterior humeral cortex fails in tension. The pull of the triceps muscle pulls the distal fragment posteriorly and proximally [3]. Traumatic nerve injury ranks the highest among all complications associated with SCH fractures. The reported risk of nerve-related complications with SCH ranges from 6.6 to 31% [4]. The anterior interosseous nerve (AIN) injury ranks the highest nerve injury associated with extension-type fractures, whereas ulnar nerve injury predominates in flexion-type neuropathy [5].

Children who present with SCH must have a complete and thorough assessment of the neurologic function of the hand to uncover any neurological deficit. Based on the patient's age, this is not always possible. Children who are four years and older can usually describe any abnormal sensation or weakness that they may have. Assessing younger children requires experience and patience. The usual symptoms of neurological injury are paraesthesia, funny feelings in some (not all fingers), loss of feeling and pain, although the latter is not discriminatory when there is a fracture. Signs may include altered or loss of sensation, loss of two points discrimination, dryness of the skin, weakness, or inability to move fingers [6].

Singer and Schorr reported for the first time on a new complication following a nerve injury in a supracondylar humeral fracture that had never been described before in the literature. They reported on self-mutilation of the fifth finger following iatrogenic ulnar nerve injury in a child with a supracondylar fracture [7]. We report here three cases of self-mutilation of index finger and thumb following median nerve injury after SCH fractures in children.

## Case presentation

Three children presented with displaced supracondylar humeral fractures after a fall. The first two were four-year-old and five-year-old boys, respectively. Median nerve injury was confirmed during the examination and before surgery. There was no vascular injury. The X-rays confirmed that they had SCH fractures of extension type.

**Figure 1:**
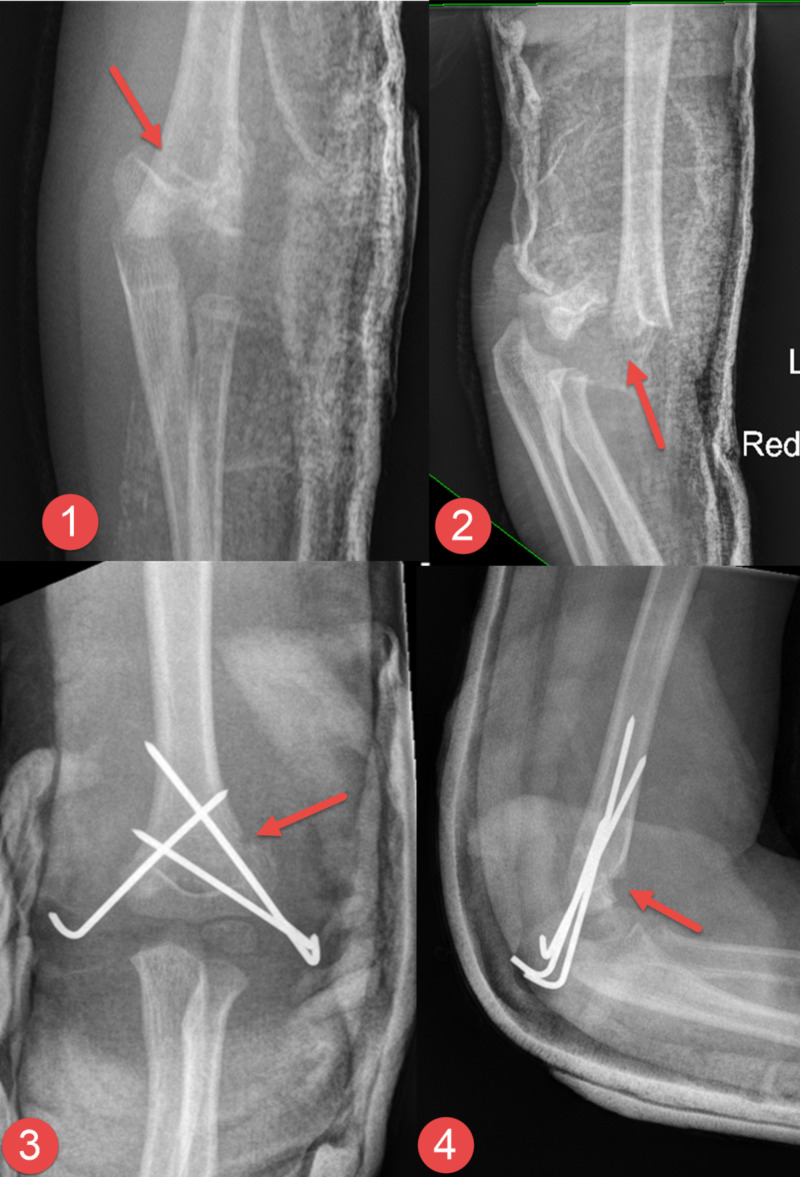
Plain radiograph of the elbow following surgery Plain radiograph of the elbow showing the pre-operative anteroposterior (AP) and lateral views (images 1 and 2, respectively) and postoperative AP and lateral views (images 3 and 4, respectively). The fracture (red arrows) was reduced and stabilized with three K-wires - two were inserted laterally and one was inserted medially.

**Figure 2:**
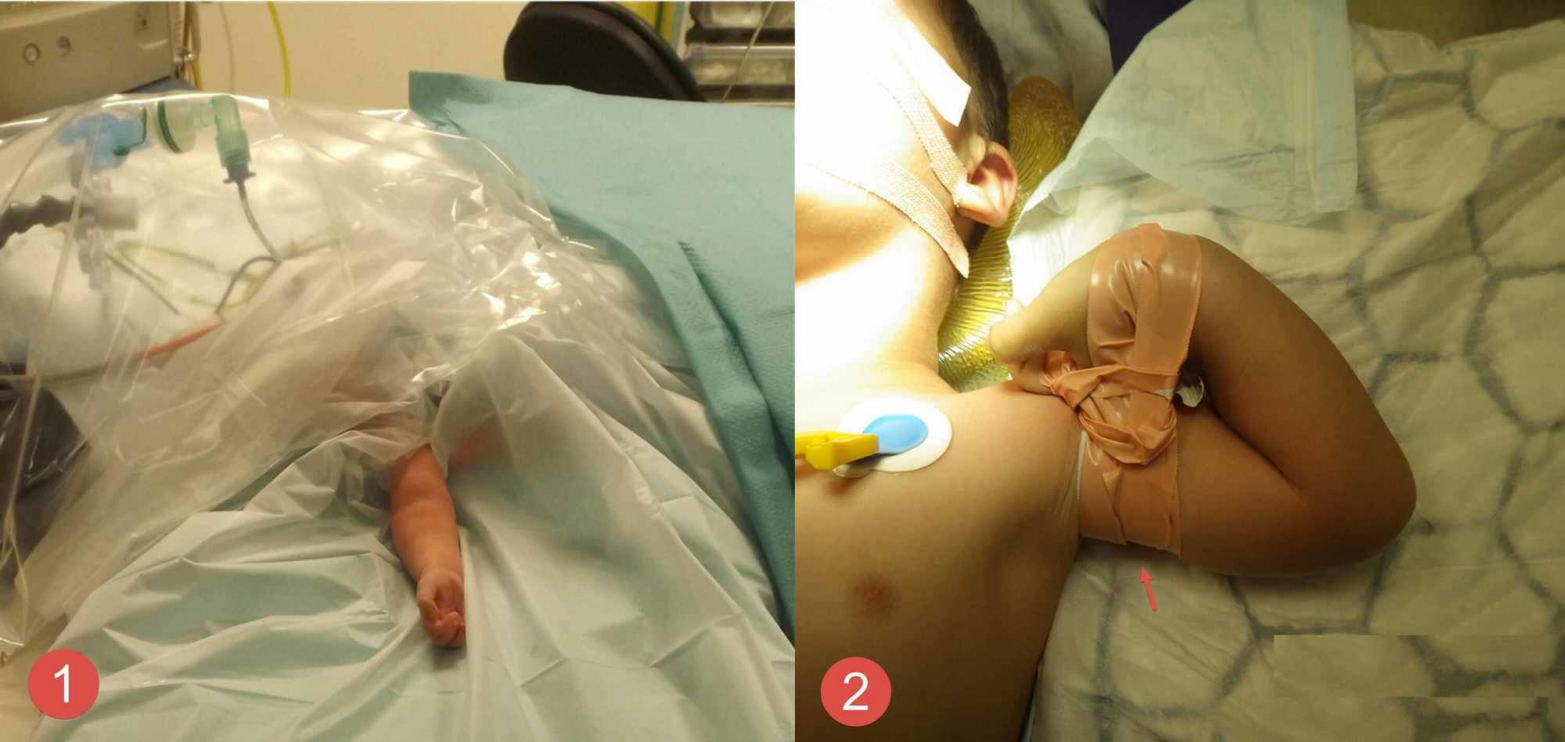
Intraoperative clinical photography Image 1 shows the upper limb preparation for reduction. No tourniquet was used. Image 2 shows the tapping of the upper limb (red arrow) in hyper-flexed position to keep the fracture reduced during the stabilisation procedure.

Patients were taken to the theatre on an urgent basis, and both fractures were reduced and stabilized using 2 mm K-wires (Figure 1). The standard technique was utilized to stabilize these fractures under general anesthesia [8]. No tourniquet was used during surgery. Closed reduction was achieved using manual traction, translation then flexion, and taping technique (Figure 2). The fluoroscopic assessment was used to confirm an acceptable reduction using anteroposterior and two oblique views at 90° to each other (lateral and medial oblique views). The lateral view was examined either by carefully rotating the elbow or by rotating the fluoroscopy. The surgical field was prepared using alcoholic chlorohexidine and draped using adhesive disposable surgical drapes. Two divergent lateral K-wires (size 2mm) were used initially to stabilize the fracture but if the fracture was still unstable, a third lateral (or medial) K-wire was depending on the fracture configuration. If the medial K-wire was used, as in Figure 1, a small incision was used to visualize and protect the ulnar nerve. The wound was closed with Vicryl Rapide™ (size 4-0).

Then the limb was placed in an above elbow back slab with the elbow in 70° of flexion. The postoperative period was uneventful, and both patients were discharged home with residual sensory symptoms in the median nerve distribution.

The third child was a six-year-old boy who presented with a supracondylar fracture following a fall. Examination revealed a badly deformed elbow and neuro-vascular deficit. His hand was cold and pale, indicating poor circulation. He had median nerve symptoms. He was taken to theatre for urgent reduction and stabilization as described above; however, the hand circulation remained poor. Therefore, the brachial artery was explored and repaired. The nerve was also explored and was found to be bruised but not transected. The hand circulation improved after artery repair. He was discharged home after a few days with residual sensory and motor symptoms.

**Figure 3:**
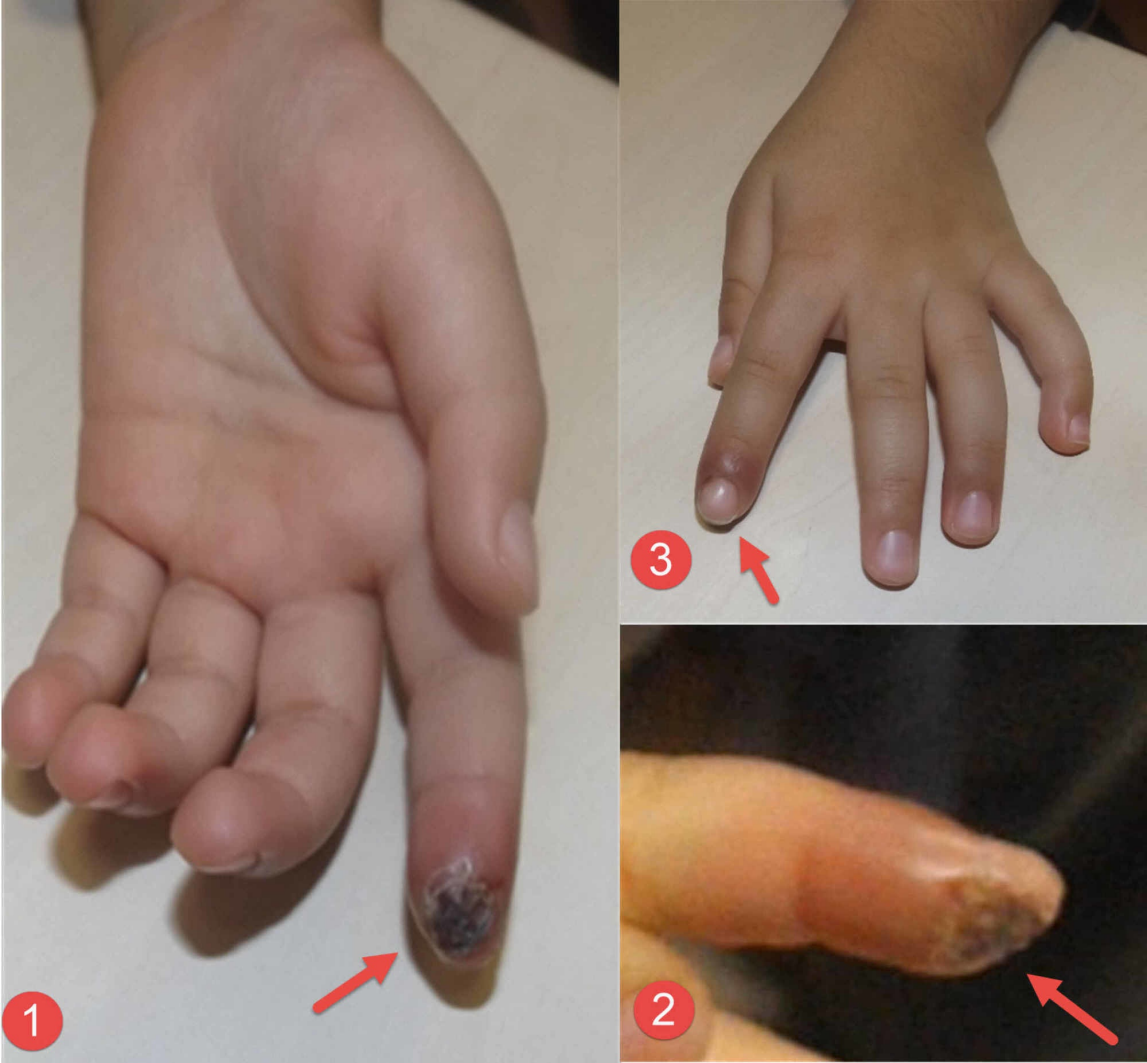
Clinical photographs of the first patient’s fingers Clinical photographs of the first patient showing the damage to the pulp of the left index finger (red arrows) from three sides - volar (image 1), side (image 2), and dorsum (image 3).

**Figure 4:**
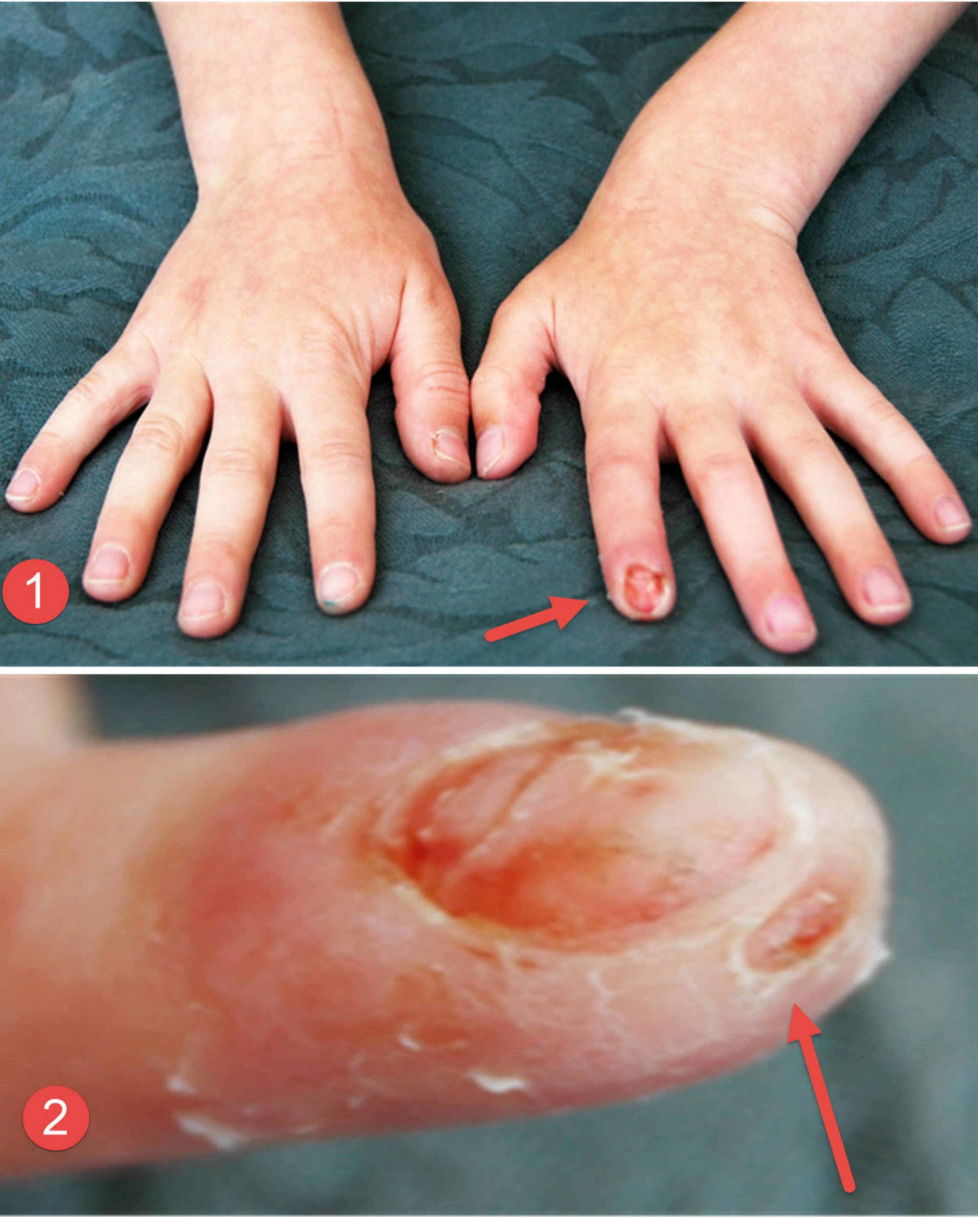
Clinical photograph of the second patient's hand Image 1 shows both hands with the obvious damage of the tip of the left index finger that was caused by biting (red arrows). Image 2 is a close-up view of the tip of the index finger showing more details of tissue damage.

**Figure 5:**
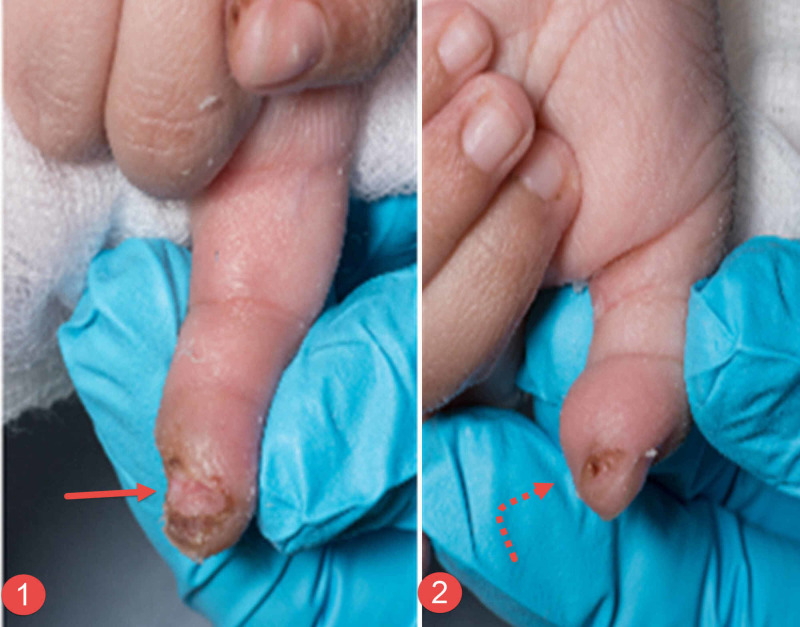
Clinical photographs of the third patient's fingers Clinical photograph of the third child showing the damage that was caused to the tips of the index finger (image 1 - straight red arrow) and thumb (image 2 - curved dashed red arrow), respectively.

In the first two weeks after surgery, parents of all three children reported that their children started biting the tip of the index fingers (Figures 3 and 4). One child was biting the thumb as well (Figure 5). This caused significant damage to the skin and soft tissues. All of them require regular dressing and oral antibiotics, but unlike the only reported case, the bite wounds were not complicated by a severe infection that necessitated amputation.

## Discussion

Minor or benign self-mutilation behaviors like nail pitting or hair pulling are common. However, severe cases are rare and usually associated with unusual congenital, hereditary, or psychiatric disorders [9-11].

In a review of 127 cases of perinatal brachial plexus injury, 4.7% of the cases had clinical evidence of self-mutilation [12]. In another study of 280 infants who were born with perinatal brachial plexus injury, 11 cases of self-mutilation behavior were identified, yielding a cumulative incidence of 3.9%. Authors were uncertain whether this behavior represented a response to pain, an exploratory response to an insensate-limb, or a response to non-painful dysesthesias [13].

Dahlin and colleagues reported on two cases of progressive self-biting of the fingers and hands that led to multiple finger amputations, in individuals of normal intelligence who displayed this behavior following spinal cord injury (SCI) [14].

Peripheral nerve injuries are common following trauma in general and fractures in particular. They require special attention to confirm the diagnosis, to monitor and intervene surgically at the right time. Some aspects of nerve injury care have not been fully delineated and new knowledge and experience are added regularly. The three patients that we presented in this study highlight another piece of information for nerve injury care and management. The altered sensation that is caused by a nerve injury makes few children bite the affected area to get some sort of relief or satisfaction.

The literature search revealed two cases only of self-mutilation of fingers following traumatic peripheral nerve injuries. The aforementioned study by Singer and Schorr who reported a one year and eight-month-old girl with supracondylar humeral fracture-flexion type. She underwent reduction and stabilization using K-wires. This resulted in an iatrogenic ulnar nerve injury. The child started self-mutilating the little finger on the affected limb, which resulted in the amputation of the distal phalanx [7].

The second case was reported in 1985. A three-years and nine-month-old girl, who had a deep laceration of the volar aspect of her hand following a fall through a glass door. No nerve injury was seen at the time of the presentation. However, four weeks after the initial presentation self-mutilation wounds were found at the tip of the second and third fingers, the child was noted to bite, scratch, and rub the two fingers occasionally [15].

To the best of our knowledge that these two cases are the first reports of self-biting of fingers following median nerve injury. It highlights the importance of awareness of the condition and the vigilance for early detection. This is particularly important in very young children who cannot express the symptoms that they experience. Protecting fingers using dressings, mallet splint or bite-averting nail polish may help to protect the fingers.

## Conclusions

Nerve injuries are common following trauma in general and fractures in particular. They require special attention to confirm the diagnosis, to monitor and intervene surgically at the right time. Self-mutilation of affected parts is a very rare complication of traumatic peripheral nerve injury that can get quickly out of control and necessities amputating the affecting parts. Patients and parents should be specifically warned about and advised to report it early. Healthcare providers should examine for in follow up appointments.
